# DNA methylation in clonal duckweed (*Lemna minor* L.) lineages reflects current and historical environmental exposures

**DOI:** 10.1111/mec.16757

**Published:** 2022-11-20

**Authors:** Morgane Van Antro, Stella Prelovsek, Slavica Ivanovic, Fleur Gawehns, Niels C. A. M. Wagemaker, Mohamed Mysara, Nele Horemans, Philippine Vergeer, Koen J. F. Verhoeven

**Affiliations:** ^1^ Department of Terrestrial Ecology Netherlands Institute of Ecology (NIOO‐KNAW) Wageningen The Netherlands; ^2^ Plant Ecology and Physiology Radboud University Nijmegen The Netherlands; ^3^ Biosphere Impact Studies Belgian Nuclear Research Centre (SCK CEN) Mol Belgium; ^4^ Wageningen University and Research (WUR) Plant Ecology and Nature Conservation Group Wageningen The Netherlands

**Keywords:** clonal reproduction, DNA methylation, epigenetic memory, *Lemna minor*, temperature stress, transgenerational

## Abstract

Environmentally induced DNA methylation variants may mediate gene expression responses to environmental changes. If such induced variants are transgenerationally stable, there is potential for expression responses to persist over multiple generations. Our current knowledge in plants, however, is almost exclusively based on studies conducted in sexually reproducing species where the majority of DNA methylation changes are subject to resetting in germlines, limiting the potential for transgenerational epigenetics stress memory. Asexual reproduction circumvents germlines, and may therefore be more conducive to long‐term inheritance of epigenetic marks. Taking advantage of the rapid clonal reproduction of the common duckweed *Lemna minor*, we hypothesize that long‐term, transgenerational stress memory from exposure to high temperature can be detected in DNA methylation profiles. Using a reduced representation bisulphite sequencing approach (epiGBS), we show that temperature stress induces DNA hypermethylation at many CG and CHG cytosine contexts but not CHH. Additionally, differential methylation in CHG context that was observed was still detected in a subset of cytosines, even after 3–12 generations of culturing in a common environment. This demonstrates a memory effect of stress reflected in the methylome and that persists over multiple clonal generations. Structural annotation revealed that this memory effect in CHG methylation was enriched in transposable elements. The observed epigenetic stress memory is probably caused by stable transgenerational persistence of temperature‐induced DNA methylation variants across clonal generations. To the extent that such epigenetic memory has functional consequences for gene expression and phenotypes, this result suggests potential for long‐term modulation of stress responses in asexual plants.

## 1 INTRODUCTION

There has been continuous interest in understanding the underlying mechanisms that allow for species to adapt in response to environmental cues. With climate change occurring at an alarming rate, a prominent question in ecology and evolutionary biology is whether or not organisms are capable of adapting to such rapid climatic changes. In the particular case of aquatic ecosystems, climate change due to anthropic activities is projected to increase mean water temperatures (IPCC, 2021). Coping with such changing environments can occur in one of two (nonmutually exclusive) ways: phenotypic plasticity (short term responses) and evolution through natural selection (Carroll et al., 2007; Hairston et al., 2005).

While genetic variation provides a fundamental basis for phenotypic differences between individuals (Sommer, 2020), epigenetic mechanisms, such as DNA methylation, are hypothesized to play a prominent role in short‐term plastic phenotypic processes of organisms. In plants, DNA methylation consists of the addition of a methyl group to cytosines, which can occur in one of three sequence contexts, CG, CHG and CHH (where H can be an A, C or T nucleotide). Depending on the cytosine context and developmental stage, DNA methylation has been linked to multiple processes, including the suppression of transposable element (TE) activity and the regulation of gene expression (Luo et al., 2018; Muyle et al., 2022; Niederhuth & Schmitz, 2017; Schmitz et al., 2019; Zhang et al., 2006). The potential of DNA methylation to regulate gene expression, associated with the fact that DNA methylation is responsive to changes in environmental conditions (Gallego‐Bartolomé, 2020; Ito et al., 2019; Liu & He, 2020; H. Zhang et al., 2018), has fuelled the idea that environmentally induced DNA methylation variants can mediate phenotypic plasticity. Moreover, because some DNA methylation variants are very stable (also across generations; Mounger et al., 2021; Wilschut et al., 2016), it has been speculated that epigenetic inheritance can sustain environmentally plastic responses over multiple generations (Bošković & Rando, 2018; Calarco et al., 2012; Hauser et al., 2011; Heard & Martienssen, 2014; Richards, 2006; van der Graaf et al., 2015). However, empirical demonstrations of these hypothesized roles of environmentally induced DNA methylation variants in (transgenerational) phenotypic plasticity have been challenging. For instance, at least part of environment‐induced DNA methylation may be but a by‐product of gene expression changes (Bewick & Schmitz, 2017; Secco et al., 2015). Furthermore, in *Arabidopsis thaliana*, the majority of environmentally induced DNA methylation variants are not transgenerationally stable (Heard & Martienssen, 2014; van Dooren et al., 2020; Wibowo et al., 2016), limiting the scope of transgenerational epigenetic inheritance.

In studying the environmental and transgenerational dynamics of DNA methylation, it is important to distinguish between the different sequence contexts of cytosine methylation. The dynamics of DNA methylation in the different sequence contexts are controlled by different enzymatic pathways, which have different accuracies in the maintenance of methylation (Niederhuth & Schmitz, 2017). This difference in enzymatic fidelity linked to the symmetry of the cytosine context on the DNA strand induces differences in environmental sensitivities and transgenerational stability of DNA methylation (Muyle et al., 2022; Niederhuth & Schmitz, 2017; van der Graaf et al., 2015). There is a growing consensus that CHH (and to a lesser extent CHG) cytosine contexts are the most responsive to environmental changes (Calarco et al., 2012; Kenchanmane Raju et al., 2019; Lu et al., 2017; Saban et al., 2020; Zhao et al., 2022). These induced CHH methylation changes are quick to revert back to their original state however, and show low transgenerational stability. In contrast, CG methylation is maintained with high fidelity between cell divisions, and therefore in turn show high transgenerational stability (Mathieu et al., 2007; Schmid, Heichinger, et al., 2018; van der Graaf et al., 2015); the involvement of CG methylation in environmental responses remains uncertain.

Currently, most knowledge on plant epigenetic inheritance comes from studies on sexually reproducing plants such as *A. thaliana* (Pecinka et al., 2009). The lack of stable inheritance of environment‐induced DNA methylation variants in sexually reproducing plants may not exclusively be due to differences in cytosine context responsiveness and stability, but could in part be explained by pigenetic reprogramming mechanisms occurring during germline formation (Feng et al., 2010; Kawashima & Berger, 2014; Schmid, Giraldo‐Fonseca, et al., 2018; Wibowo et al., 2016). This reprogramming can include the (partial) erasure and re‐establishment of epigenetic marks between generations. Yet a very large number of plants (including agricultural crops) propagate clonally and thus do not depend on germline formation to reproduce. Hence, it has been proposed that asexually reproducing plants may show higher and longer stability of environmentally sensitive epigenetic variants across clonal generations (Dong et al., 2019; Douhovnikoff & Dodd, 2014; Verhoeven & Preite, 2014). While marker‐based studies have indicated persistence of DNA methylation patterns from one clonal generation to the next (Rendina González et al., 2018; Wilschut et al., 2016), the development of comprehensive, high‐resolution techniques targeting species with (such as WGBS and RRBS) and without (epiGBS, epiRAD and bsRADseq; Schield et al., 2016; Trucchi et al., 2016; van Gurp et al., 2016) a reference genome, now allow for more comprehensive and substantive studies to be conducted.

Here, we present a reduced‐representation bisulfite sequencing analysis (epiGBS) of DNA methylation in the highly clonal common duckweed, *Lemna minor*, after episodes of heat stress. *L. minor*, belonging to the family Lemnaceae, is a prominent macrophyte and is known for its rapid clonal growth, with a doubling time of ~2 days (Ziegler et al., 2015) resulting in genetically uniform populations. With its fast reproduction time, small size and ease of manipulation, the genus *Lemna* is widely used in laboratory conditions for both physiological and ecotoxicological studies (Aliferis et al., 2009; Lee et al., 2021). Recently, with the development of sequencing technology and availability of genomic tools, *L. minor* has also become a species of interest for genetic and epigenetics studies. Indeed, recent efforts have developed genomic and transcriptomic resources for the species (Mardanov et al., 2008; Van Hoeck et al., 2015).

Taking advantage of the rapid clonal reproduction of *L. minor*, we tested whether exposure to high temperatures produces transgenerational memory in the DNA methylation profiles of *L. minor* individuals. In order to induce changes in DNA methylation and mimic changes in temperature in aquatic environments due to climate change (McCaw et al., 2020), we exposed genetically identical lineages to different (24°C, 30°C or fluctuating) temperature regimes for a period of 6 weeks. After these 6 weeks, each lineage was subsequently cultured for an additional 3 weeks at both 24 and 30°C. Over several time points, frond area and number were measured. Furthermore, DNA methylation screening was conducted once in all lineages at the end of the experiment, after 3 weeks growth in the 24 or 30°C environment (corresponding to 3–12 clonal generations). Using this experimental design, the specific hypotheses tested on the environmental responsiveness of DNA methylation in *L. minor* were: (i) What is the effect of high temperature exposure to DNA methylation profiles? (ii) Do DNA methylation profiles show a memory effect of temperature treatments experienced multiple clonal generations ago? (iii) Is the DNA methylation response to high temperature different when lineages have themselves been previously exposed to high temperatures multiple clonal generations ago? We demonstrate that high‐temperature stress leaves a transgenerational footprint that is detectable in DNA methylation profiles (specifically in CHG contexts) many clonal generations after removal of the stress environment. This transgenerational change in DNA methylation profiles due to stress suggests that long‐term epigenetic stress memory occurs in clonally reproducing plant lineages.

## 2 MATERIAL AND METHODS

### 2.1 *Lemna minor* stock population

*Lemna minor* individuals (serial no. 1007; ID no. 5500) were provided by Dr Nele Horemans' laboratory from the Belgian Nuclear Research Center (SCK‐CEN). In order to allow for a more in‐depth understanding of *L. minor*'s response to temperature, a single genotype was used. A stock population was obtained by aseptically culturing the individuals in 100 ml of Hunter's nutrient medium (Brain & Solomon, 2007) in cotton‐plugged 250‐ml Erlenmeyer flasks. The flasks were stored in growth cabinets at constant temperatures (24 ± 0.2°C) and light (100 ± 10 μmol m^−2^ s^−1^) which are the standard culturing conditions for *L. minor in* ecotoxicological tests (OECD, 2006). The stock population was maintained, prior to the experiment, by aseptically transferring triple‐fronded individuals every 14 days into fresh nutrient medium. This weekly transfer was done in order to limit nutrient stress due to depleted medium as well to avoid populations experiencing overcrowding within their flasks.

### 2.2 Experimental design

**FIGURE 1:**
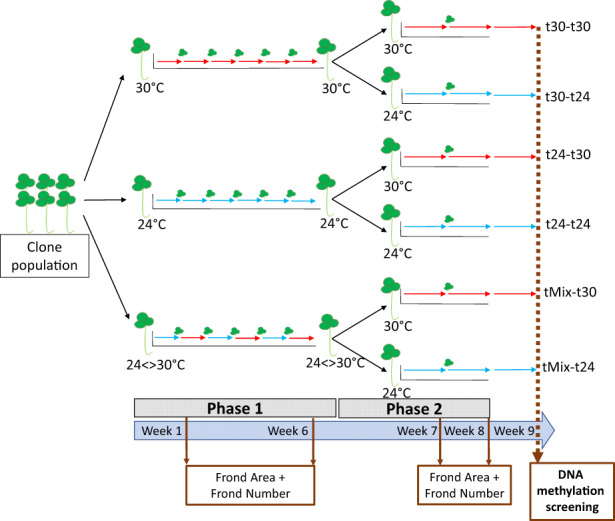
Design of the two‐phase temperature exposure experiment. Starting from a clonal *Lemna minor* founder population, individual plants were used to establish replicated, genetically uniform lineages that were exposed to different temperature treatments. Phase 1 (weeks 1–6): Cloned lineages were maintained either at 24°C (t24), 30°C (t30) or at weekly fluctuating 24↔30°C (tMix). Phase 2 (weeks 7 and 8): Each phase 1 lineage was maintained at both 24°C (t24) and 30°C (t30). During the entire experiment (weeks 1–8), weekly transfers to fresh medium were done by transferring a single individual; from week 8 to 9 all individuals were transferred to obtain sufficient material for DNA extraction. Growth phenotypes were measured after weeks 1, 6, 7 and 8. DNA methylation screening of each lineage was measured only once at the end of phase 2 using epiGBS after week 9.

To establish a cloned population, one single founder individual was selected from the stock population and allowed to propagate for 14 days. From this cloned population, individuals were taken to establish genetically uniform replicated lineages that were exposed to different experimental temperature treatments. The experiment was conducted in two phases: Phase 1 where lineages were exposed to different temperature treatments; and Phase 2 where lineages were then grown and evaluated in two contrasting common temperature environments (see Figure 1 for experimental design overview).

#### 2.2.1 Phase 1

Forty‐eight cloned lineages were maintained at three different temperature regimes for 6 weeks (16 replicate lineages per temperature): a controlled temperature environment of 24°C, a high but nonlethal temperature environment of 30°C (Kuehdorf & Appenroth, 2012; Vymazal, 2008) and a weekly fluctuating temperature (24°C↔30°C) environment. The fluctuating temperature treatment was included because episodic exposure to a stressful environment may trigger different responses than continuous exposure (Kronholm & Ketola, 2018; Wibowo et al., 2016). Each week, per lineage, one triple‐fronded individual was aseptically transferred into freshly prepared nutrient medium and placed back into its respective temperature regime. To avoid the same individual from being transferred for multiple consecutive weeks, the transferred individual was marked using a small plastic ring. Nevertheless, due to the nature of *L. minor*'s rapid growth rate, we are unable to accurately know which generation was transferred weekly. Assuming a clonal doubling time of ~2 days (Ziegler et al., 2015), within 1 week the transferred individual multiplies into a population consisting of individuals that are theoretically 1–4 clonal generations removed from the founder, irrespective of treatment. At the end of the 6‐week Phase 1 period, individuals were thus at least six and maximally 24 clonal generations removed from the founder individual at the start of phase 1.

#### 2.2.2 Phase 2

A 2‐week testing phase directly followed the Phase 1 temperature treatments. From each of the 48 Phase 1 lineages, one triple‐fronded individual was placed at 24°C and one at 30°C, with weekly transfers as described above. At the end of Phase 2, per lineage, all individuals were transferred into freshly prepared nutrient medium and grown for an additional 7 days in their respective temperature regime, obtaining enough plant material for subsequent DNA extraction. At the moment of sampling, 3 weeks after the end of Phase 1 and assuming a clonal generation time of ~2 days, populations consisted of a mixture of individuals of 3–12 clonal generations removed from the founder individual at the start of Phase 2.

Overall, at the end of both phases, the two‐factor crossed experimental design should have resulted in six treatment groups with 16 independent replicate lineages each: t24‐t24, t24‐t30, t30‐t24, t30‐t30, tMix‐t24, tMix‐t30 (indicated by the different experimental Phase 1–Phase 2 temperature treatments). However, due to some lineages perishing during the experiment, between 12 and 15 replicate samples per experimental group were sampled (14 t24‐t24; 12 t24‐t30; 13 t30‐t24; 12 t30‐t30; 15 tMix‐t24; 12 tMix‐t30) (Table S1).

### 2.3 Phenotypic measurements

At the start and end of Phase 1 (after week 1 and week 6), frond number and frond area were measured. The same phenotypes were measured, on a weekly basis, during Phase 2 (week 7 and week 8). Pictures of fronds were taken with a Sony Cyber‐shot Digital camera DSC‐RW100 at a fixed distance (7 cm) from the growing surface. From these pictures, frond number was determined using imagej (Schneider et al., 2012), with the total frond area being calculated using winfolia (Lobet, 2017). Within the Erlenmeyer flasks used to grow the clonal populations, a plastic strip of fixed length (1.80 cm) was floated as a scale for measurement calibration.

When statistical test assumptions were met (normality of residuals and homogeneity of variances), differences in either frond number or frond area for each measured week were analysed using a one‐way ANOVA (for weeks 1 and 6; testing for a Phase 1 temperature effect on phenotypes) or a two‐way ANOVA model (for weeks 7 and 8; testing for a Phase 1 and Phase 2 temperature effect on phenotype). We included the Phase1 × Phase2 interaction term in the two‐way ANOVA to test if the response to the current Phase 2 temperature regime is dependent on the temperature experienced by the previous generation during Phase 1. The *p*‐values were calculated using the *aov* function (r stats Package, version 3.6.2). For weeks 6 and 8 of frond number and weeks 6, 7 and 8 of frond area, model validation revealed heteroscedasticity of variances. In such cases, a linear regression with generalized least squares extension was used (Pinheiro & Bates, 2006; West et al., 2006) which uses variance–covariate terms to allow for unequal variance. The *p*‐values were calculated using the *gls* function (r nlme Package, version 3.1‐152).

### 2.4 epiGBS library construction

#### 2.4.1 Sampling and DNA extraction

Unlike measuring phenotypic traits, screening for DNA methylation patterns of each lineage was done only once, at the end of Phase 2 (Figure 1). Sampling of frond tissue for DNA analysis consisted of the removal of all roots, ensuring that only frond material was collected. DNA methylation is tissue‐specific, with roots having different methylation patterns compared to shoots in plants (Widman et al., 2014; Zhang et al., 2011). To ensure that enough DNA material was obtained and to limit potential individual plant effects due to differences in developmental stages, about 30 fully developed triple‐fronded individuals were pooled and flash‐frozen in liquid nitrogen as a single sample. Samples were stored at −80°C until further analysis. Samples were homogenized using a Qiagen TissueLyser II with the use of two stainless steel beads (45 s at a frequency of 30.00 s^−1^). DNA isolation was performed using the Macherey‐Nagel NucleoSpin Plant II kit. Optimal quality and quantity of DNA was obtained using the cell lysis Buffer PL2 provided by the kit. After DNA extraction, all samples were diluted to 30 ng μl^−1^ of DNA.

#### 2.4.2 epiGBS library preparation

An adapted version of the epiGBS protocol (van Gurp et al., 2016) was followed, as described by Gawehns et al. (2022). In brief, after full randomization of all samples, DNA was digested with DNA methylation‐insensitive restriction enzymes *Ase*I and *Nsi*I (ensuring that the enzymes did not induce a bias by cutting primarily in [non‐]methylated regions of the genome). Hemimethylated adapter pairs were then ligated to the digested DNA, with each adapter containing a 4‐ to 6‐nucleotide sample‐specific barcode, followed by a string of three random nucleotides (NNN) (known as unique molecular identifier [UMI]) and an unmethylated cytosine (used to annotate Watson and Crick strands, as well as to estimate the bisulphite conversion rate). Samples were then multiplexed together, concentrated and cleaned of smaller fragments (<60 bp) using the NucleoSpin Gel & PCR cleanup Kit protocol. Size selection was done using 0.8× SPRIselect magnetic beads, selecting for DNA fragments of 300 bp and lower. The nicks induced by the use of hemimethylated adapters were then repaired through the use of dNTPs that contain 5‐meC's resulting in fully ligated and methylated adapters. Multiplexed samples were bisulphite converted using the EZ DNA Methylation‐Lightning kits, following the manufacturer's protocol. The converted DNA was PCR (polymerase chain reaction)‐amplified before a final DNA concentration, PCR clean‐up and size‐selection step. The final library was sequenced paired‐end (PE 2 × 150 bp) with a 12% phiX spike on one lane using an Illumina HiSeq X sequencer.

#### 2.4.3 epiGBS pipeline

Sequencing data were analysed using the epiGBS2 pipeline (epiGBS2 commit: 5a70433fa; Gawehns et al., 2022), using the “de novo” option with default parameters (95% sequence identity in the last clustering step and a clustering depth threshold of one) to generate experiment‐specific local genomic references for the epiGBS reads. In brief, the epiGBS2 pipeline first removes all PCR duplicates based on the identity of the UMI inserted in the adapter sequences. The UMI ensures that only true PCR clones, and not biological duplicates, are removed from the sequencing data. Using the stacks 2 software (Catchen et al., 2013), samples are then demultiplexed according to their barcodes. Using a consensus de novo reference generated from the experimental data, the pipeline then maps the sequence fragments using the alignment program star. From these mapped sequences, both methylation and single nucleotide polymorphism (SNP) variants were called, using epiGBS custom scripts. The called methylation sites were reported in a methylation. bed file format.

For downstream analysis, the methylation. bed file was filtered as follows: first, one sample that showed a very low number of total reads was removed from the data (Sample 1_2 belonging to the t24‐t30 treatment group). Next, a minimum 10× coverage threshold was applied, as well as excluding from the analysis the 0.1% of sites with the highest coverage (in this data set: all cytosines with a coverage >1148 reads). The coverage distribution of all reads before and after filtering can be found in Figures S1 and S2. Subsequently, only cytosines which were present in at least 80% of all samples (irrespective of the temperature regime) were considered in the analysis.

#### 2.4.4 epiGBS loci—General overview

The library for the 80 samples generated 831,669,476 raw sequencing reads of which 625,769,980 (75.2%) were successfully demultiplexed and assigned to individual samples. The de novo assembly resulted in 146,745 clusters of 32–290 bp long (average = 207), with an average of 10.2 fragments making ‐up one contiguous cluster (min = 1; max = 2359). The sum of all contig lengths amounted to 281,866 bp. Assuming that epiGBS fragments do not overlap and that the published reference assembly of *L. minor* covers the entire genome, this would mean that epiGBS fragments capture a maximum of 5.86% of the whole *L. minor* genome. The bisulphite conversion rate was estimated at 98.19%, based on the number of correctly bisulphite‐converted control cytosines found within the adapters. After filtering, as described above, 932,871 cytosines (9.55%) were retained for further analysis. Given that DNA methylation can occur within three cytosine contexts in plants (CG, CHG and CHH), with each context having different properties and different functions (Zhang et al., 2018), all analyses are done separately for each context. Within the retained cytosines, 111,695 were found in the CG context, 97,501 in the CHG context and 723,675 in the CHH context.

### 2.5 Annotation

De novo epiGBS reference sequences were mapped against the annotated *L. minor* genome (available in the CoGe database with ID 27408; Van Hoeck et al., 2015) using the bowtie tool (Langmead et al., 2009). The generated bam files were then converted into bed files using bedtools bamtobed functionality (Quinlan & Hall, 2010). Through these steps, epiGBS fragments were classified as either landing within or near a gene (maximum of 1000 bp downstream), within an intergenic region or in an unannotated region. Since the annotation of *L. minor* reference genome does not possess TE information, the same epiGBS reference fragments were also run through repeatmasker (Embryophyta as reference species collection; version 4.0.6) (Smit et al., 2015), obtaining homologous TE information. The obtained epiGBS annotation was used to determine in which genomic features (gene, intergenic, TEs or unannotated) differentially methylated cytosines or epiGBS loci were located.

### 2.6 DNA methylation screening

#### 2.6.1 Global methylation levels

Global methylation levels, for all cytosines combined but also for each cytosine context (CG, CHG and CHH) separately, were calculated as the average per‐cytosine methylation level for each individual sample. After confirming that assumptions of normality and homogeneous variance were met, differences in global methylation levels between experimental groups were tested using a two‐way ANOVA model (*aov* function, stats r package version 3.6.2) followed by post hoc pairwise comparisons (*emmean_test* function, rstatix r package version 0.7.0). The two‐way ANOVA provides overall tests of Phase 1 temperature effects and Phase 2 temperature effects as well as the interaction between the two temperature phases. The Phase 1 main effect tests whether experimental groups that experienced different Phase 1 temperatures (t24 vs. t30 vs. tMix) show different methylation levels. The Phase 2 main effect tests whether experimental groups that experienced different Phase 2 temperatures (t24 vs. t30) show different methylation levels. A significant Phase 1 effect is interpreted as a transgenerational memory effect while a significant Phase 2 effect is interpreted as an effect of current temperatures of the lineages. A significant interaction term indicates that the methylation responses to temperature in Phase 2 are dependent on the temperature regime experienced by the previous lineages during Phase 1.

#### 2.6.2 Principal component analysis and redundancy analysis

Principal component analysis (PCA) was done, per cytosine context and after removal of cytosines with missing data, to visualize patterns of DNA methylation variation between experimental groups. After removal of missing data, 897,931 cytosines remained (107,406 CG, 93,306 CHG and 697,219 CHH). Several redundancy analyses (RDAs) were done to assess the significance of observed differences between experimental groups in overall methylome patterns (*rda* function, vegan r package, version 2.5‐7) by using permutation testing (*anoca.cca* function, vegan r package, version 2.4‐2, nperm = 999). One RDA was conducted to test Phase 2 temperature effects, comparing Phase 2 T24 to T30 groups for each cytosine context. Additionally, RDA was then performed to test Phase 1 temperature effects separately for each Phase 2 group (T24 or T30). Effects were considered significant at *p* < .05.

#### 2.6.3 Differentially methylated cytosines (DMCs)

To identify DMCs, beta‐binomial regression models were fit in r using DSS (dss r package, version 2.38; Y. Park & Wu, 2016), using arcsine link function, Wald testing and without smoothing. The two‐factor crossed experimental design was modelled through a linear model framework, allowing us to test for effects of Phase 1 temperature, Phase 2 temperature and their interaction on methylation levels of individual cytosines detected at the end of Phase 2. Significant Phase 2 effects identify cytosines that are responsive to the current temperature treatment, while significant Phase 1 effects identify cytosines whose methylation show a memory effect of temperature treatment experienced several clonal generations ago. A significant interaction indicates that the DNA methylation responses to the current Phase 2 temperature treatment are dependent on the temperature treatment experienced by previous clonal generations during Phase 1. The *p*‐values for each factor were adjusted for multiple testing using a false discovery rate (FDR) threshold of 0.05. Per‐cytosine differences in DNA methylation between experimental groups were calculated by subtracting the average per‐cytosine methylation level over all samples of one temperature treatment from that of another temperature treatment. To further refine the list of potentially relevant DMCs, a methylation difference of 20 percentage points or higher was applied for some analyses as an additional selection threshold. Using the obtained annotation, an enrichment analysis was performed to determine if DMCs were overrepresented in specific genomic features. Using a hypergeometric test, also known as Fisher's exact test (*phyper* function, stats r package, version 3.6.2), a genomic feature was considered significantly enriched in DMCs, after correcting for multiple testing, at an FDR threshold of <0.05. Fold‐change was calculated using the following equation:

$$\frac{\left(\#\text{DMCs in genomic feature}/\text{Total}\#\text{DMCs}\right)}{\left(\#\text{Cytosines in genomic feature}/\text{Total}\#\text{Cytosines}\right)}.$$

#### 2.6.4 Differentially methylated epiGBS loci

Due to the short nature of epiGBS fragments (ranging between 32 and 290 bp with median length of 181 bp), no formal tests for differentially methylated regions (DMRs) were performed. We considered epiGBS loci as differentially methylated when they possessed a minimum of 10 statistically significant DMCs (irrespective of the absolute methylation difference between cytosines), either for individual cytosine contexts (only CG, CHG or CHH DMCs) or loci for which a mixture of cytosine contexts (combination of CG, CHG and CHH DMCs).

## 3 RESULTS

### 3.1 Phenotypic measurements

**FIGURE 2:**
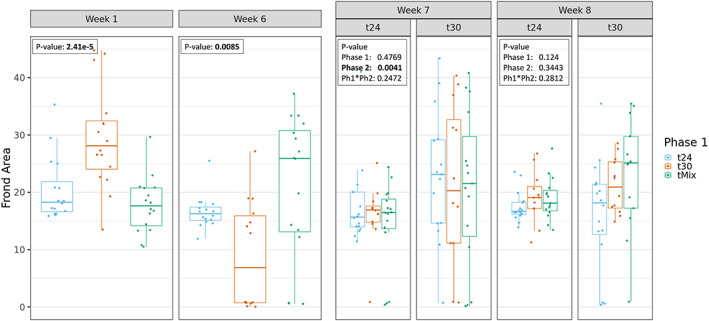
Total frond area of all *Lemna minor* individuals per lineage measured after 1, 6, 7 and 8 weeks of temperature treatment. Weeks 1 and 6 represent phase 1 of the experiment where lineages where exposed to either 24°C (t24), 30°C (t30) or weekly fluctuating temperature of 24↔30°C (tMix). Weeks 7 and 8 represent phase 2 of the experiment where lineages were then placed in a common environment of either 24 or 30°C.

**FIGURE 3:**
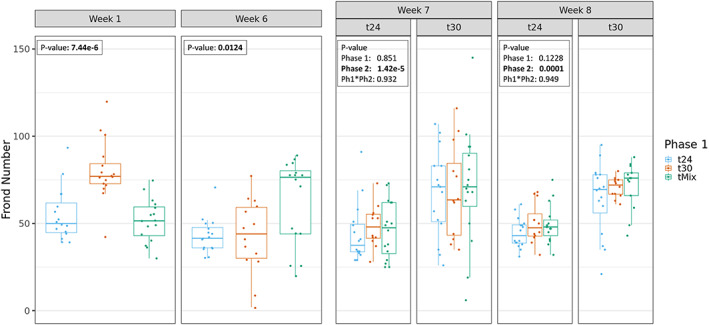
Total number of fronds counted within each *Lemna minor* lineages measured after 1, 6, 7 and 8 weeks of temperature treatment. Weeks 1 and 6 represent phase 1 of the experiment where lineages where exposed to either 24°C (t24), 30°C (t30) or weekly fluctuating temperature of 24↔30°C (tMix). Weeks 7 and 8 represent phase 2 of the experiment where lineages were then placed in a common environment of either 24 or 30°C. Asterisks show which treatments were significantly different (*p* < .05).

During Phase 1, significant temperature effects were observed for both frond area and frond number (Figures 2 and 3). Exposure to 30°C resulted in an initial growth increase (week 1) both in average frond number and area compared to the T24 treatment group. This positive effect on growth was not maintained after prolonged exposure (week 6), when negative effects of high temperature were observed, as reduced average frond area compared to t24. This decrease in average frond area seemed to be caused by some replicate lineages showing strongly reduced growth while other lineages maintained growth rates similar to that at 24°C (Figure 2). Plants grown in the TMix environment were exposed to 24°C in week 1 and thus showed similar phenotypes as the T24 group in that week. In week 6, TMix populations were grown at 30°C and showed higher frond number and area compared to the T24 and T30 groups.

Week 7 indicates the beginning of Phase 2. Effects of Phase 2 temperatures were significant for both average frond area and number after week 7 of growth in one of the two common temperature regimes (Figures 2 and 3) with only frond number being significantly different after week 8. When significant, lineages currently found at 30°C had systematically higher average frond number and area compared to populations currently grown at 24°C. No significant Phase 1 transgenerational effect as well as no interaction effect between Phase 1 and Phase 2 treatments was detected for both measured phenotypes.

### 3.2 Global methylation levels

**FIGURE 4:**
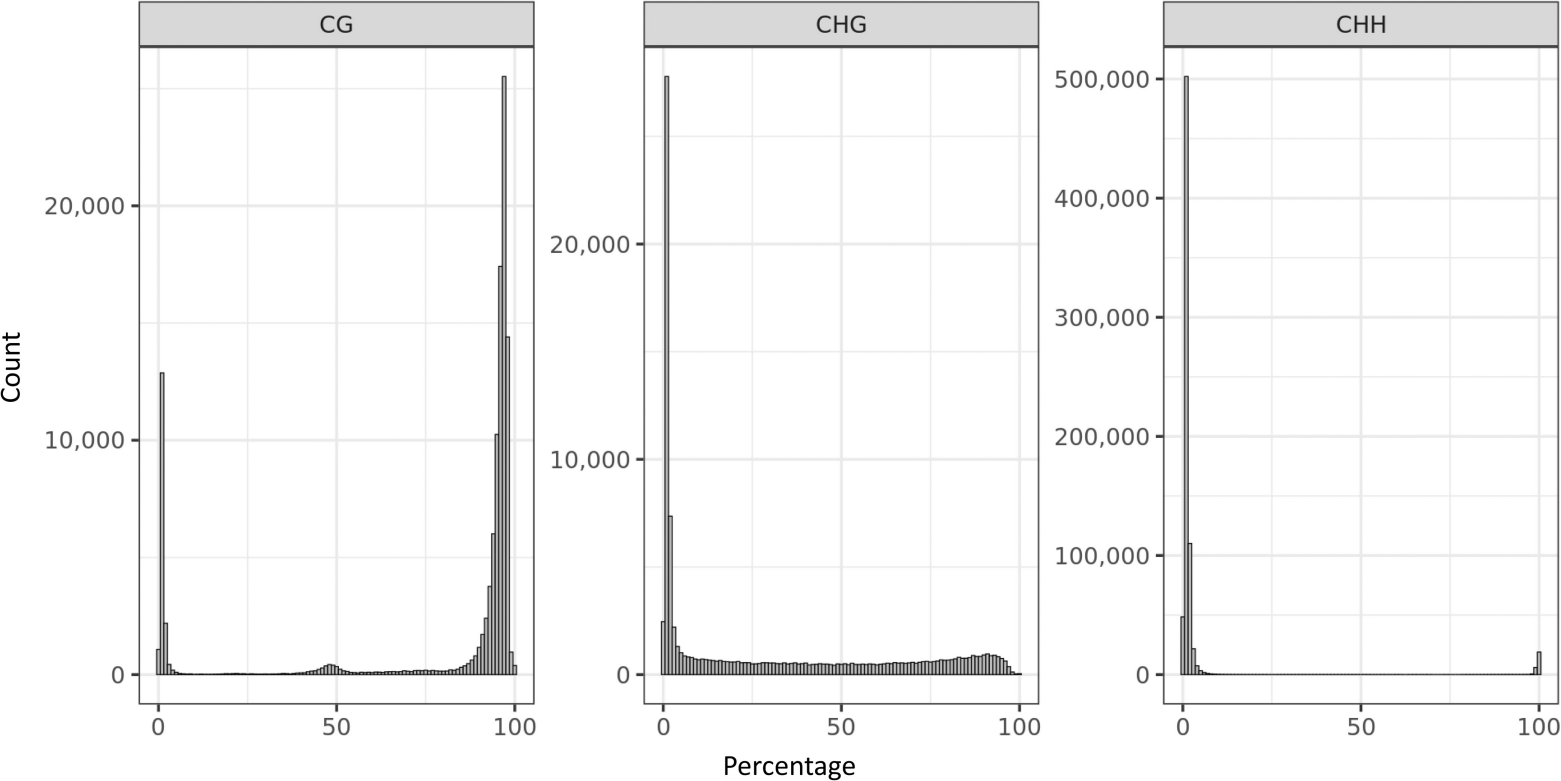
DNA methylation landscape of *Lemna minor*. Histogram of DNA methylation percentage of cytosines in CG, CHG and CHH context, calculated as the mean value across all samples of per‐cytosine methylation level estimates.

Average methylation levels varied per cytosine context. In CG context, the average cytosine methylation level, across all samples, was 78.9%, with global methylation patterns showing a bimodal distribution with strong bias towards high methylation levels (Figure 4). Cytosines in CHH context showed opposite patterns, with low average methylation levels of 4.76%, and ~80% of CHHs being unmethylated. In CHG context, cytosines showed average methylation levels of 30.43%, with a bias to low methylation (~50% of cytosines with methylation level <10%) but otherwise showing a relatively uniform distribution above 10%.

**FIGURE 5:**
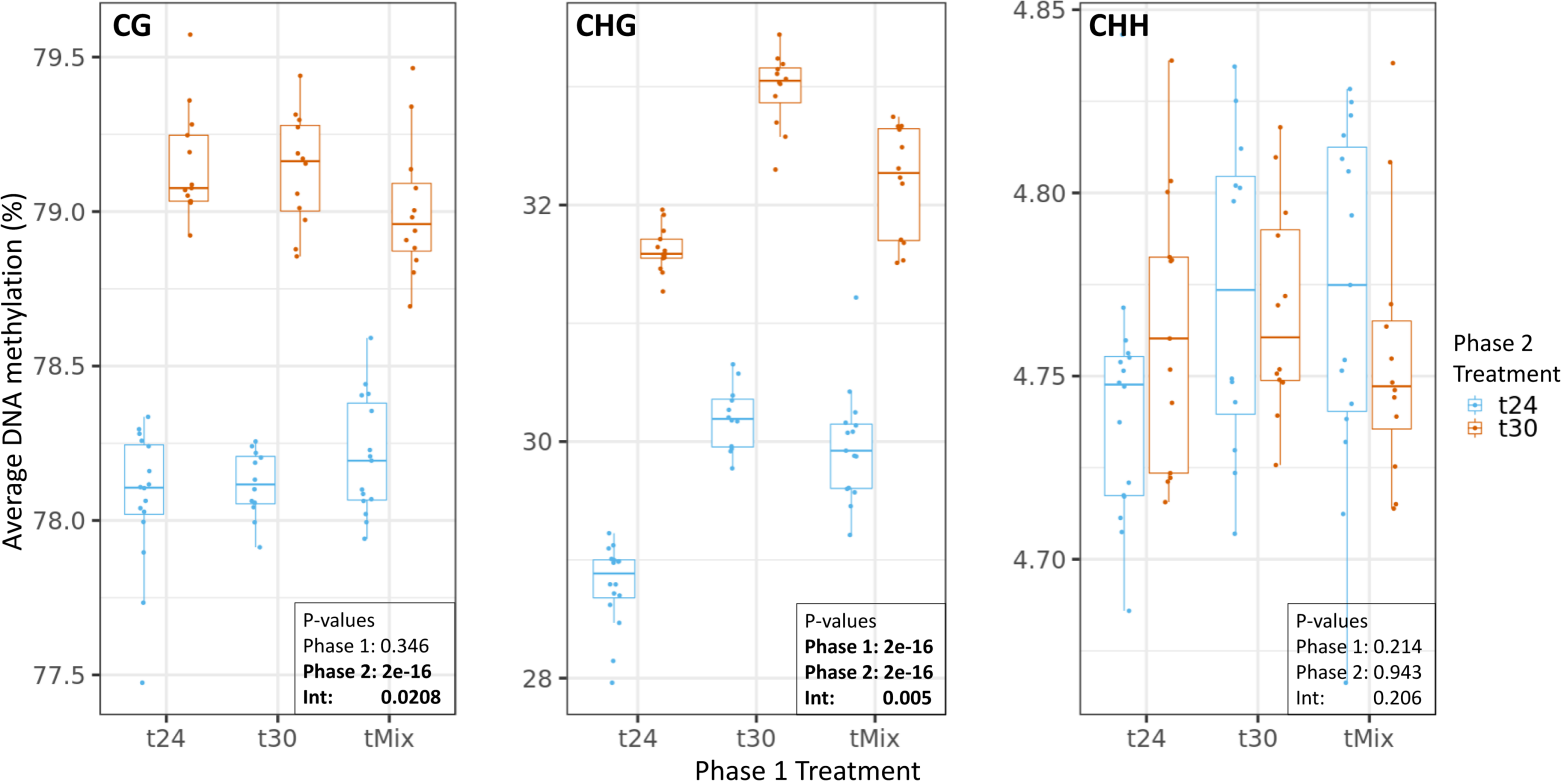
Average per‐cytosine DNA methylation level in temperature treatments. During phase 1, lineages were exposed to either 24°C, 30°C or weekly fluctuating temperature of 24↔30°C (tMix). In phase 2, lineages were then placed in a common environment of either 24 or 30°C. Panels show results for cytosines in different sequence context (CG, CHG, CHH). Each point in the graph represents an independent experimental lineage.

Global methylation levels were responsive to temperature treatments and cytosine context. An overall increase in cytosine methylation level was observed in Phase 2 t30 compared to t24 in both CG and CHG contexts, but not in CHH context (Figure 5). A significant memory effect of the ancestral (Phase 1) temperature treatment was also detected in CHG context: lineages that had experienced the 30°C treatment in Phase 1, either continuously or episodically, showed higher DNA methylation levels at the end of Phase 2 compared to lineages that experienced 24°C in Phase 1. Furthermore, a significant interaction effect was detected in CG and CHG, indicating that at the global methylation level, methylation patterns induced by the current temperature regimes (Phase 2) are conditional on the temperature regimes experienced by the lineages during Phase 1.

**FIGURE 6:**
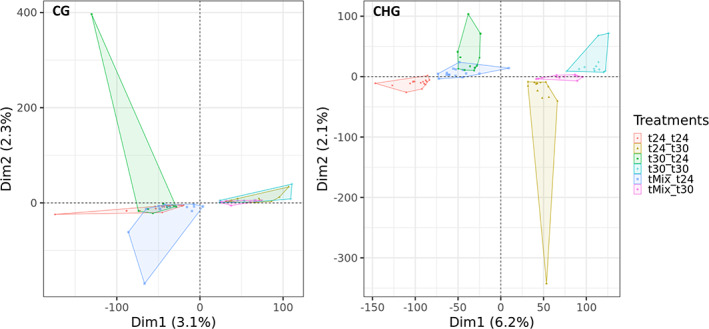
Changes in DNA methylation profiles due to temperature treatments, for methylation in CG and CHG cytosine context. During phase 1, lineages were exposed to either 24°C, 30°C or weekly fluctuating temperature of 24↔30°C (tMix). In phase 2, lineages were then placed in a common environment of either 24 or 30°C. Each point in the graph represents an independent experimental lineage.

To further characterize patterns of temperature‐induced changes in DNA methylation, a PCA was used to visualize, for each cytosine context, the variation induced (factominer r package, version 2.4). Although the variation explained by the first PC axes was relatively low (3.1% for CG, 6.2% for CHG and 2.1% for CHH), RDA revealed that DNA methylation profiles in CG and CHG contexts showed significant Phase 2 temperature effects (*p* < .001 in both cases). A clear clustering was observed of samples grown continuously in either high 30°C temperature or control 24°C temperature (Figure 6), signifying strong current environment effects in CG and CHG methylation profiles. No temperature effects were detected in CHH context. Strikingly, and in addition to a Phase 2 temperature effect, methylation in the CHG context showed a significant Phase 1 temperature effect: within the Phase 2 clusters, three different Phase 1 subclusters can be distinguished that were significantly differentiated (RDA of Phase 1 effect within Phase 2 groups: *p* < .01 within both the T24 and T30 groups) (Figure 6). Thus, CHG methylation showed a memory effect of temperatures experienced many clonal generations ago. The pattern of sample clustering based on methylation in CHG context suggests partial, but incomplete, reversal of 30°C‐induced DNA methylation changes (Phase 1) during growth at 24°C in Phase 2: maximum separation of clusters is visible between t24‐t24 and t30‐t30 samples, while the t30‐t24 samples are becoming more similar again to, but are still separated from, the t24‐t24 samples. While this indicates that the environment experienced by the ancestral lineages can leave a memory footprint in the DNA methylation profile of the current lineages, it also suggests a reversal of methylation states at individual loci from the 30°C‐induced state to the 24°C control state.

### 3.3 Differentially methylated cytosines

**FIGURE 7:**
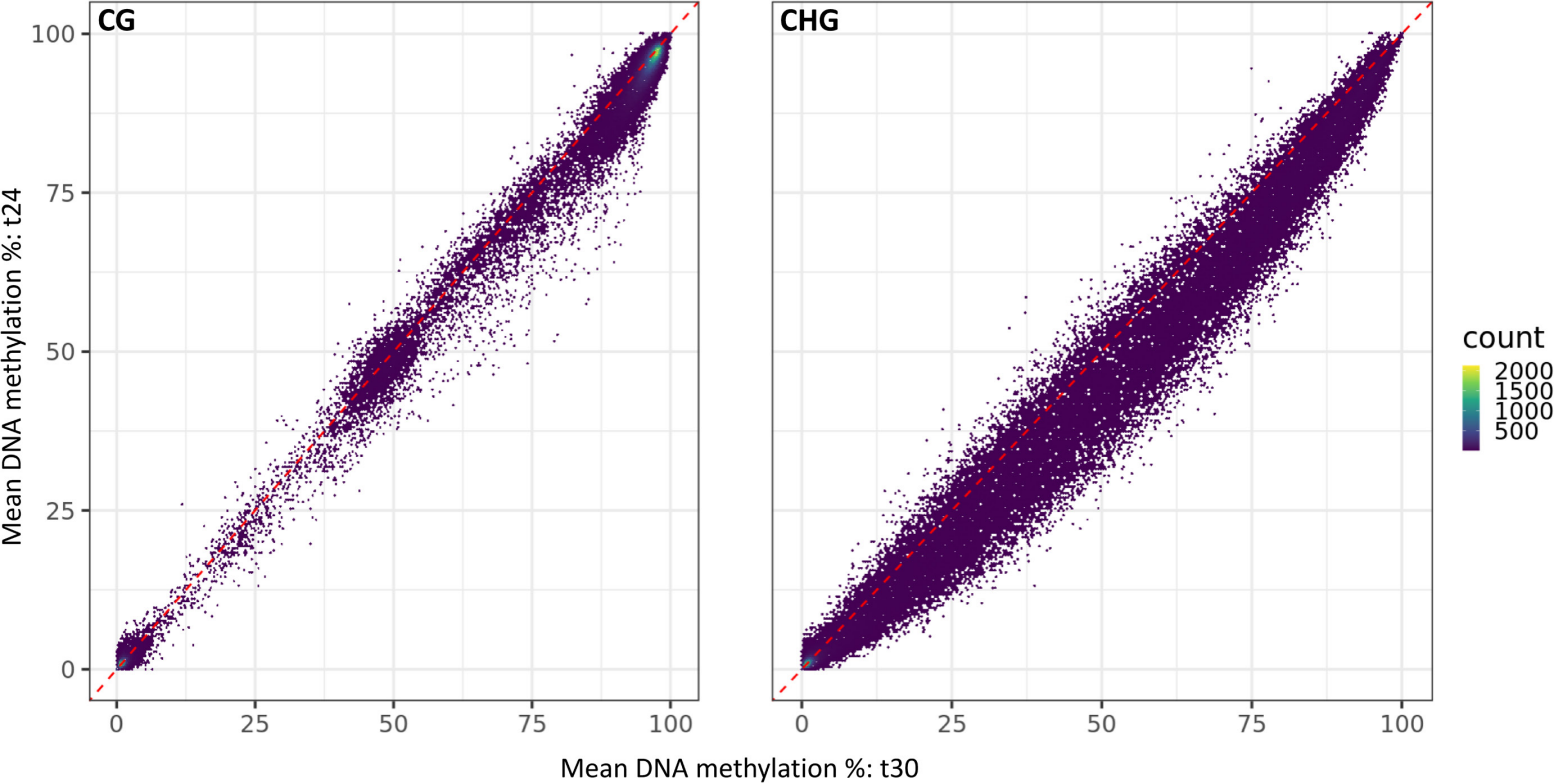
Scatter plot of cytosine methylation percentage at 24°C vs. 30°C, for cytosines in CG and CHG context. For each cytosine, methylation level (%) was calculated for both temperature treatments as the average methylation level of all samples from phase 2.

As seen in Tables 1, 41,496 DMCs were affected by the Phase 2 temperature regime (9328 CG DMCs, 32,164 CHG DMCs and four CHH DMCs). These DMCs indicate that a very large number of cytosines showed a DNA methylation response to high temperature exposure: almost 7.4% of tested cytosines in CG context and 28.7% of tested cytosines in CHG context showed a statistically significant effect of Phase 2 temperatures, even after correcting for multiple testing. Most changes are relatively small and in the direction of hypermethylation at 30°C (Figure 7). The highly replicated nature of the experiment (up to 15 replicate lineages per experimental group) presumably provided very high statistical power to detect subtle changes as statistically significant. To validate the DMCs obtained, the data were also analysed using a different analysis approach: a logistic regression (using Methylkit, methylkit r package, version 3.12), which yielded similar absolute numbers of DMCs due to Phase 2 temperature effects (CHG context: 28,008 DMCs using DSS and 26,010 DMCs using Methylkit, of which 25,479 overlap; CG context: 8260 DMCs using DSS and 7259 DMCs using Methylkit, of which 6551 overlap; Figure S3).

**TABLE 1:** Number of differentially methylated cytosines (DMCs) due to phase 1 and phase 2 temperature effects, by genomic feature and sequence context

	All cytosines	Significant DMCs					Strongly responding DMCs			
		Phase 1		t24‐t24 vs. t30‐t24 contrast	Phase 2		Phase 1		Phase 2	
		No. of DMCs	Fold ∆	No. of DCMs	No. of DMCs	Fold ∆	No. of DMCs	Fold ∆	No. of DMCs	Fold ∆
**Gene**										
CG	5020	0	*—*	0	313	0.813	0	*—*	3	2.421
CHG	4499	37	0.892	59	607	0.448	13	1.286	5	1.032
CHH	29,473	0	*—*	0	0	*—*	0	*—*	0	*—*
**Intergenic**										
CG	67,190	0	*—*	0	4667	0.905	0	*—*	24	1.447*
CHG	62,590	547	0.948	783	15,244	0.809	123	0.875	56	0.831
CHH	437,298	0	*—*	0	0	*—*	0	*—*	0	*—*
**TE**										
CG	12,160	0	*—*	0	1106	1.185*	0	*—*	0	*—*
	106,809	128	1.214*	166	4422	1.285*	43	1.674*	16	1.300*
CHH	793,597	0	*—*	0	0	*—*	0	*—*	0	*—*
**Unannotated**										
CG	37,206	0	*—*	0	3242	1.136*	0	*—*	3	0.327
CHG	28,291	273	1.046	348	11,891	1.396*	61	0.959	38	1.247
CHH	247,549	0	*—*	1	4	3.206*	0	*—*	1	3.206*
**Total**										
CG	121,576	0		0	9328		0		30	
CHG	106,809	985		1356	32,164		240		115	
CHH	793,597	0		1	4		0		1	

*Note*: Classification by structural annotation was done for all identified methylated cytosines, for all significant (*p* < .05) DMCs and for the subset of strongest‐responding DMCs (significant DMCs with a methylation difference of 20 percentage points or higher between treatments; identified using a cross‐factorial model) as well as significant (FDR < 0.05) DMCs identified by contrasting the t24‐t24 to the t30‐t24 lineages. Fold ∆ represents the fold enrichment ratio. A significant fold increase was calculated using a hypergeometric test and is indicated with an asterisk (*). During Phase 1 lineages were exposed to either 24°C, 30°C or a weekly alternation of 24↔30°C. During Phase 2, lineages were then placed in a common environment of either 24 or 30°C.

In CHG context (but not CG or CHH), 985 cytosines showed a significant Phase 1 effect, reflecting a transgenerational stress memory. In total, 937 out of these 985 (95.2%) Phase 1 DMCs also show a Phase 2 effect on DNA methylation (Figure S4). In other words, the cytosines that express a significant memory effect of past temperature treatment are a subset of the cytosines that are temperature‐sensitive to begin with. This supports the idea that the Phase 1 memory effect is caused by transgenerational stability, over several clonal generations, of temperature‐induced DNA methylation changes that were triggered during the Phase 1 treatment. Consistently, a strong signal is detected when testing the DNA methylation difference between t24‐t24 and t30‐t24 lineages (1356 DMCs in CHG context, tested as an a priori contrast within the Phase 1 factor of the full factorial model, see Table 1), which is expected if the Phase 1 memory effect is caused by long‐term persistence of DNA methylation modifications that were induced during Phase 1 30°C treatment. No significant interaction effect of Phase 1 × Phase 2 temperature treatment was detected at the individual cytosine level.

The majority of DMCs induced by the Phase 2 temperature treatment (~50%) were found within intergenic regions, with around 10% landing in TEs, and only a small fraction landing in or within a gene. The hypogeometric test revealed significant enrichment of CG and CHG Phase 2 DMCs as well as CHG Phase 1 DMCs in TEs only (Table 1). Filtering DMCs to include only the strongest‐responding cytosines (a minimum of 20 percentage point cytosine methylation difference between any of the experimental groups) drastically reduced the number of DMCs. Interestingly, of the DMCs showing a Phase 1 temperature effect in CHG context, a relatively large proportion belonged to this category of strongly responding cytosines (249 out of 985 significant Phase 1 DMCs; see Table 1) indicating that long‐lasting transgenerational stress memory in CHG methylation is associated with strong methylation differences. Furthermore, within these strongly responding DMCs, enrichment was observed within TEs.

### 3.4 Differentially methylated epiGBS loci

Consistent with the DMC results, most of the epiGBS loci that contained 10 or more DMCs (“putative DMRs”) were observed in CHG context, in response to the current Phase 2 temperature treatment and within intergenic or unannotated genome regions (Figure S5). Four putative DMRs were observed that showed a Phase 1 temperature effect (transgenerational memory) and these were all very strongly responding DMRs, with all DMCs within these DMRs being 20 percentage points or more differentially methylated. A total of 483 putative DMRs were observed in response to current (Phase 2) temperature treatments (30 DMRs consisting of solely CG DMCs, 245 of CHG DMRs, zero of CHH DMRs and 204 for DMRs consisting of combined cytosine contexts; Figure S5). Of these, 15 landed within or near a gene (five CG, five CHG and five all cytosine context combined). A functional annotation of these 15 genes was conducted, (Table S2), with four genes of these 15 encoding proteins either involved in gene expression regulation or involved in response to heat stress. *MED33A* and *BFA2* code for proteins involved in the regulation of RNA polymerase II, a multiprotein complex required for gene transcription (Bonawitz et al., 2012). *SDR1* codes for a protein that is involved in the abscisic acid biosynthesis process. Abscisic acid is a plant hormone important for the plant's response to environmental stresses, including heat stress (Islam et al., 2018) and *BG2* is involved in the response to cold (Amme et al., 2006).

## 4 DISCUSSION

In this study, we present DNA methylation and phenotypic data acquired from a highly clonal aquatic species, the common duckweed *Lemna minor*, after experimental exposure to either control, high or fluctuating temperature growing conditions. Using epiGBS we showed that high temperature induces many changes in DNA methylation in both CG and CHG context. More importantly, in a subset of the responsive CHG cytosines, DNA methylation levels showed a memory of temperature treatments experienced several clonal generations ago. Structural annotation of the epiGBS loci showed that methylation changes are enriched within TEs. The observation of an epigenetic footprint of environments experienced many generations ago is in contrast to what has been reported previously in several sexually reproducing plant species (Pecinka et al., 2009; Wibowo et al., 2016), but in accordance with our hypothesis under asexual reproduction, and suggests that a subset of environment‐induced DNA methylation variants is be transgenerationally stable for multiple clonal generations.

Numerous studies have shown that changes in the environment can influence DNA methylation patterns. However, there seems to be high variation between species and stresses in the exact nature of the DNA methylation response (Mirouze & Paszkowski, 2011; Niederhuth et al., 2016; Sahu et al., 2013). In *Arabidopsis*, which has been the model species for many DNA methylation studies in plants, gene body‐like DNA methylation occurs primarily in the CG context, while repeats and TEs can show DNA methylation in all three types of cytosine context (Chan et al., 2005; Dubin et al., 2015). When exposed to environmental stressors, DNA methylation changes often occur in the CHH context, with less frequent and extreme variation occurring in the CG and CHG contexts (Dubin et al., 2015; Sun et al., 2021; Xu et al., 2018; Yaish et al., 2018). The patterns observed in duckweed are different: CHG and CG are the primary cytosine contexts whose methylation level responds to stress exposure, not CHH. In the closely related *Spirodela polyrhiza*, partial and complete loss of genes involved in the DNA methylation pathway have been demonstrated (Harkess et al., 2020). This loss has led to a drastic decrease in genome‐wide methylation in all three cytosine contexts, and especially in CHH context (~10% CG, ~3.3% CHG and ~0.1% CHH) as well as a loss in CG gene‐body methylation compared to what is normally observed in land plants. In *L. minor*, the methylation levels obtained in this experiment show much higher levels (78.9% CG, 30.4% CHG and 4.76% CHH), very much comparable to ranges measured in other plant species (Niederhuth et al., 2016). Furthermore, upon blasting the protein sequence of the main proteins involved in the DNA methylation pathway (MET1, DCL1, DCL3, AGO4, CMT3, DRM1, DRM2, NRPB1, CMT1, RDM1, RDR1), we found strong homology with all of the targeted protein sequences within the transcriptome of *L. minor* (Table S3). All of these factors imply a unique loss of DNA methylation levels in *S. polyrhiza* only, and thus the absence of an *L. minor* stress response in CHH context is not due to absence of CHH methylation. The observed specific patterns of CG‐ and CHG‐biased DNA methylation response to environmental changes in *L. minor* have rarely been documented in other plant species (Bewick & Schmitz, 2017; Takuno et al., 2016). We speculate that in the case of this experiment the duration of the multigenerational stress might explain these observed differences: CHH may show a rapid response to environmental change, but after continued, multigenerational exposure to the altered environment CHH might no longer respond to the new and now constant environment. A multigenerational stress experiment in *Arabidopsis* exposed to different gamma radiation levels for three generations found similar results, with DMRs predominantly found in CG context and to a lesser extent in CHG context, with no effect observed in CHH cytosine context (Laanen et al., 2021).

The main aim of this study was to determine if a transgenerational memory of heat stress could be detected in *L. minor*'s DNA methylation profiles, even once the stress is removed. Such a legacy effect was observed in CHG DNA methylation (as seen in Table 1) 3 weeks after removal of the heat stress in a subset of heat‐affected cytosines, with partial but incomplete reversal of induced patterns towards control 24°C patterns. This pattern is consistent with the hypothesis that plants which reproduce without a germline undergo reduced epigenetic resetting, allowing for strong transgenerational stability of spontaneous and environmentally induced DNA methylation variants (Kinoshita & Jacobsen, 2012; Verhoeven & Preite, 2014). Similar results were observed in the clonal alligator weed *Alternanthera philoxeroides* (Shi et al., 2019). In this experiment, using vegetative cuttings, Methylation‐Sensitive‐AFLP revealed that stress‐induced DNA methylation patterns were maintained for over 10 clonal generations, with progressive reversal of these patterns back towards a common state. We point out that our experimental design did not track DNA methylation changes during the course of the experiment, so no direct evidence is provided at the level of individual loci that stress exposure in Phase 1 of the experiment triggered DNA methylation modifications that were subsequently stably transmitted into Phase 2. Instead, we measured DNA methylation at the end of Phase 2 and detected evidence of Phase 1 temperature effects on DNA methylation. This proves that a Phase 1 treatment effect has carried over to the end of Phase 2. We suggest that stable transmission of Phase 1 treatment‐induced DNA methylation variants (i.e., absence of resetting between clonal generations) is a probable explanation. Several observations from our data support this hypothesis of stable transgenerational inheritance. First, the PCA patterns based on CHG methylation show that continuous exposure to heat stress induces strong and directional changes in the current methylation profiles. Yet when placing lineages grown at 30°C (during Phase 1) into 24°C (during Phase 2), the methylome profile of the lineages are intermediate compared to lineages constantly grown at 24 or 30°C. Thus, after removing the high temperature stress, the methylome profiles are shifting back towards the methylation state of lineages constantly grown at 24°C. This suggests reversal of methylation level of individual loci from the 30°C‐induced state to the 24°C state across a large number of clonal generations. Second, we showed that cytosines that express a significant memory effect of past Phase 1 temperatures are a subset of the cytosines that are temperature‐sensitive during Phase 2. This observation is most easily explained by stable inheritance across generations of 30°C‐induced DMCs. Nevertheless, to confirm whether these observed patterns indeed show stable inheritance of DNA methylation marks across a clonal generation, a time‐series approach would be required, where methylation levels of individual loci can be tracked across the different generations.

Our study was not designed to demonstrate functional consequences of DNA methylation variants; this would require, at the very least, insight into gene expression effects of the treatments. However, the absence of significant long‐lasting transgenerational memory at the level of frond number and frond area could indicate that transgenerational epigenetic inheritance does not play a role in adaptive phenotypic plasticity. Yet, a true lack of phenotypic effects is difficult to confirm based on our results. Previous studies have shown that the adaptive interpretation of phenotypic variation in *L. minor* is quite complex (Vasseur & Aarssen, 1992a, 1992b; Wilkinson, 1963). This complex interpretation is also the case in our study, with frond area and number showing dynamic changes over time, with individuals increasing or decreasing in frond number and area depending on the duration of the high‐temperature stress. Furthermore, frond number and area represent performance traits, whereas adaptive phenotypic plasticity may target underlying functional traits (such as root length, biomass or chlorophyll fluorescence) whose regulation may function to maintain performance homeostasis across environments (Ruprecht et al., 2014). Screening for molecular phenotypes, such as gene expression, would be a logical subsequent step to evaluate if inherited methylation variants have functional relevance, as gene expression should show the most direct link to DNA methylation effects.

One other striking result from our study is the large proportion of cytosines affected by the temperature stress. For instance, 28.7% of the cytosines in CHG context were significantly differentially methylated due to the current Phase 2 temperature treatment. While DNA methylation responses to heat stress have been reported in other species to target specific functional pathways that are relevant to stress responses (Korotko et al., 2021), consistent with the hypothesis that induced DNA methylation variants can mediate phenotypic plasticity via gene expression regulation, in the case of *L. minor* these genome‐wide changes in methylation levels are clearly not restricted to a few functional loci. We note that much of the DNA methylation variation in plant genomes is not expected to have functional consequences for gene expression, for instance because DNA methylation can build‐up as a by‐product of gene expression itself (Secco et al., 2015) or as presumably neutral epimutations in transcribed genes (Wendte et al., 2019). Unravelling a potential functional role of induced DNA methylation would therefore involve a search for a (potentially small) subset of loci that matter among many changes that could be nonfunctional. While we identified few putative DMRs near or within a gene which might hint at functional regulation of processes related to temperature tolerance (in particular *MED33A*, *SDR1*, *BFA2* and *BG2*), a clear result of our study is that methylation responses are enriched in TEs; it is unclear if this has functional consequences for gene expression (Baduel & Colot, 2021). Nevertheless, this enrichment of TEs is consistent with previous studies in other plant species which have shown that environmental stresses influence the DNA methylation state of TEs (Baduel & Colot, 2021; Matzke & Mosher, 2014; Wibowo et al., 2016). Possibly, the cumulative effect of methylation changes at a large number of TEs might explain the genome‐wide methylation response that we observed.

Our study clearly demonstrates that a subset of environmentally induced CHG DNA methylation variants can show strong memory effects of a stress that was experienced many clonal generations ago. This stable and long‐lasting memory provides evidence that some form of molecular information has been inherited across clonal generations, with transgenerational stability of induced DNA methylation variants a strong candidate mechanism to explain our observations. It is an open question if, or to what extent, such stable methylation variants have functional consequences for gene expression. If some do, this could hint at the role DNA methylation has in mediating long‐term transgenerational plastic responses. One can speculate that adding such a transgenerational dimension to the plant's repertoire of plastic responses might be of evolutionary benefit to clonal lineages, which in the absence of genetic adaptation need to rely heavily on phenotypic plasticity to cope with environment heterogeneity (Baker, 1965; Parker et al., 1977).

## AUTHOR CONTRIBUTIONS

KJFV and SP designed the experiment. SP, SI and MVA conducted the greenhouse and laboratory aspect of the experiment. NH provided stock *L. minor* individuals. CAMW provided training and necessary material for epiGBS. Statistical analysis was performed by MVA, MM and PV. MVA, KJFV and PV drafted the manuscript, with input and approval of the final version by all authors.

## CONFLICT OF INTEREST

The authors have no conflict of interest to declare.

### OPEN RESEARCH BADGES

This article has earned an Open Data badge for making publicly available the digitally‐shareable data necessary to reproduce the reported results. The data is available at [https://doi.org/10.5281/Zenodo.5680942].

## Supporting information


Appendix S1

## Data Availability

The raw sequencing data and demultiplexed sequencing data have been deposited in NCBI (BioProject no. PRJNA883550). The filtered methylation data, epiGBS de novo reference, as well as all phenotypic data we deposited in Zenodo (Van Antro et al., 2022a, 2022b; DOI 10.5281/Zenodo.5680942). All analysis R scripts and the epiGBS pipeline scripts corresponding to the pipeline version used to obtain the methylation data have been made publicly accessible via gitlab: https://gitlab.bioinf.nioo.knaw.nl/MorganeA/script‐for‐l.minor‐paper.git.
