# Supplementary material for: DNA methylation in clonal duckweed (Lemna minor L.) lineages reflects current and historical environmental exposures
Source: Mol Ecol. 2022 Nov 20;32(2):428–43. doi: 10.1111/mec.16757 (PMC10100429; doi:10.1111/mec.16757)
Supplement: Supplementary file 1 — Appendix S1 [file MEC-32-428-s001.pdf]

MOLECULAR ECOLOGY

Supplemental Information for:

DNA methylation in clonal Duckweed lineages (*Lemna minor* L.) reflects current and historical environmental exposures

Morgane Van Antro, Stella Prelovsek, Slavica Ivanovic, Fleur Gawehns, Niels C.A.M Wagemaker, Mohamed Mysara, Nele Horemans, Philippine Vergeer, Koen J.F Verhoeven

Table of Contents:

**Supplementary Figure 1**	Page 2
**Supplementary Figure 2**	Page 3
**Supplementary Figure 3**	Page 4
**Supplementary Figure 4**	Page 5
**Supplementary Figure 5**	Page 5
**Supplementary Table 1**	Page 6
**Supplementary Table 2**	Page 8
**Supplementary Table 3**	Page 9

MOLECULAR ECOLOGY

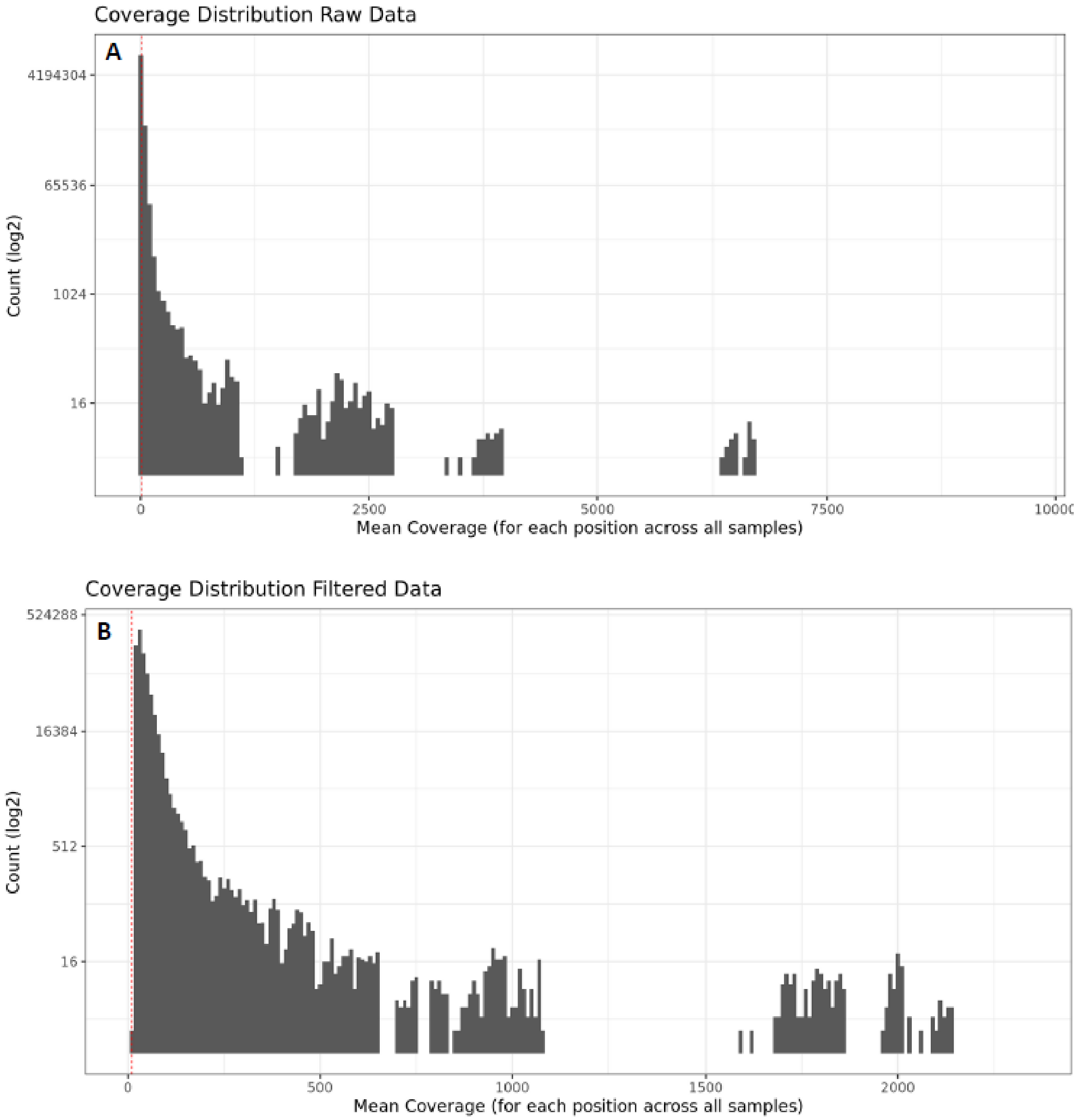

Supplementary Figure 1: Mean Coverage distribution of individual cytosines across all samples A) before and B) after filtering. Raw sequencing data was filtered as follow: a minimum 10x coverage threshold was applied (represented by the red dotted line), as well as excluding from the analysis the 0.001% sites with the highest coverage. Subsequently, only cytosines which were present in at least 80% of all samples (irrespective of the temperature regime) were considered in the analysis.

MOLECULAR ECOLOGY

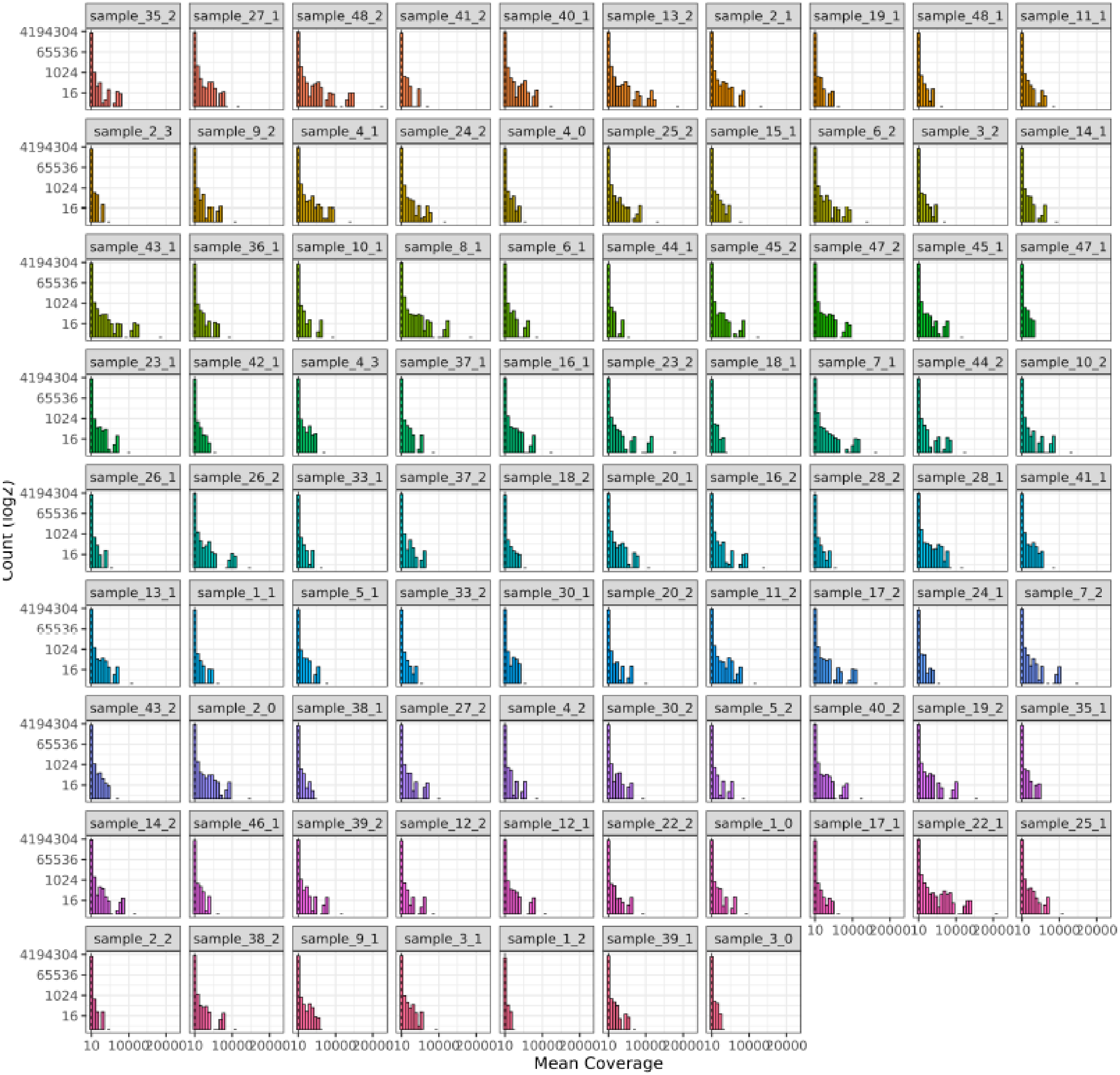

Supplementary Figure 2: Mean Coverage distribution of individual cytosines and samples after filtering. Raw sequencing data was filtered as follow: a minimum 10x coverage threshold was applied (represented by the red dotted line), as well as excluding from the analysis the 0.001% sites with the highest coverage. Subsequently, only cytosines which were present in at least 80% of all samples (irrespective of the temperature regime) were considered in the analysis.

MOLECULAR ECOLOGY

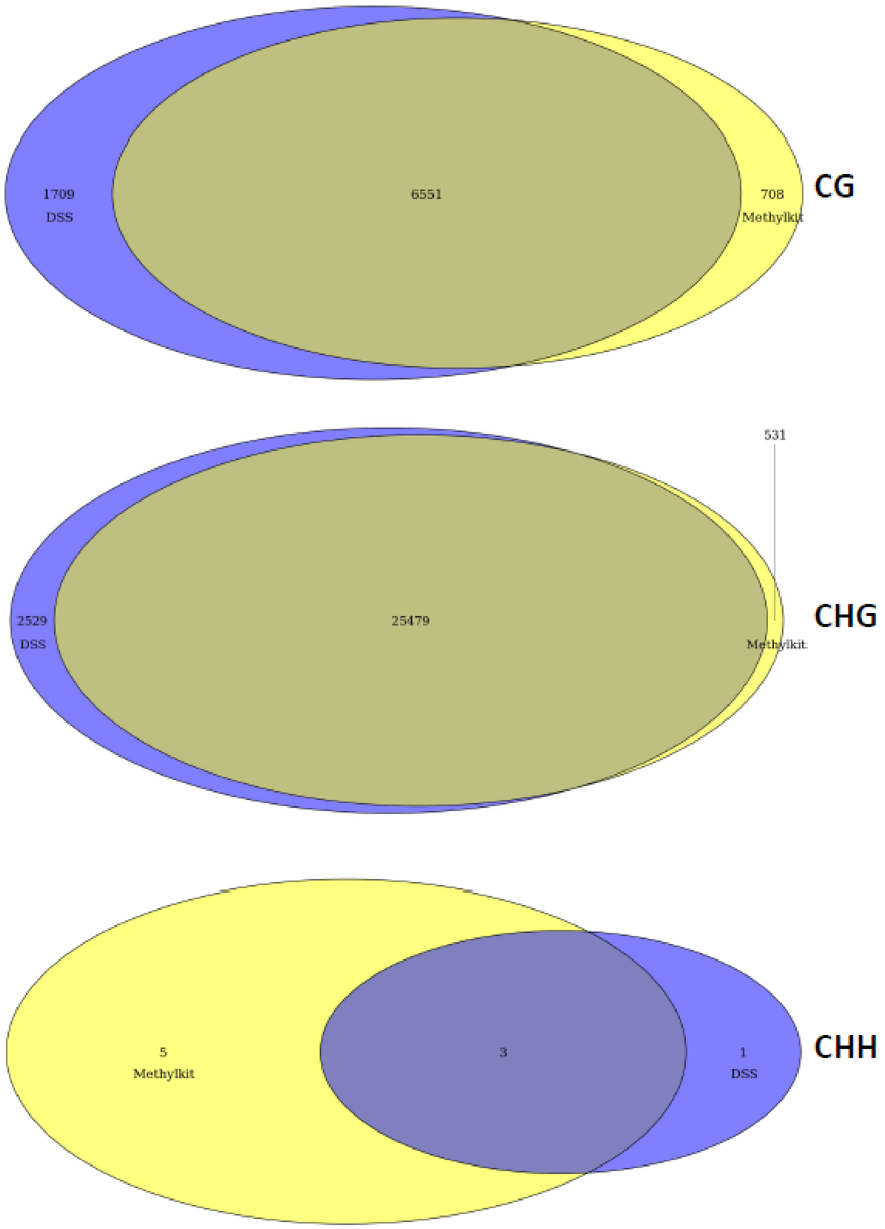

Supplementary Figure 3: **Comparison of detected DMCs between DSS and MethylKit** for phase 2 cytosines in the CG, CHG and CHH context. In purple are DMCs identified using DSS but were not detected by MethylKit. In Yellow are DMCs identified by MethylKit but not by DSS while intermediate colours show DMCs which were identified by both detection tools and thus are shared.

MOLECULAR ECOLOGY

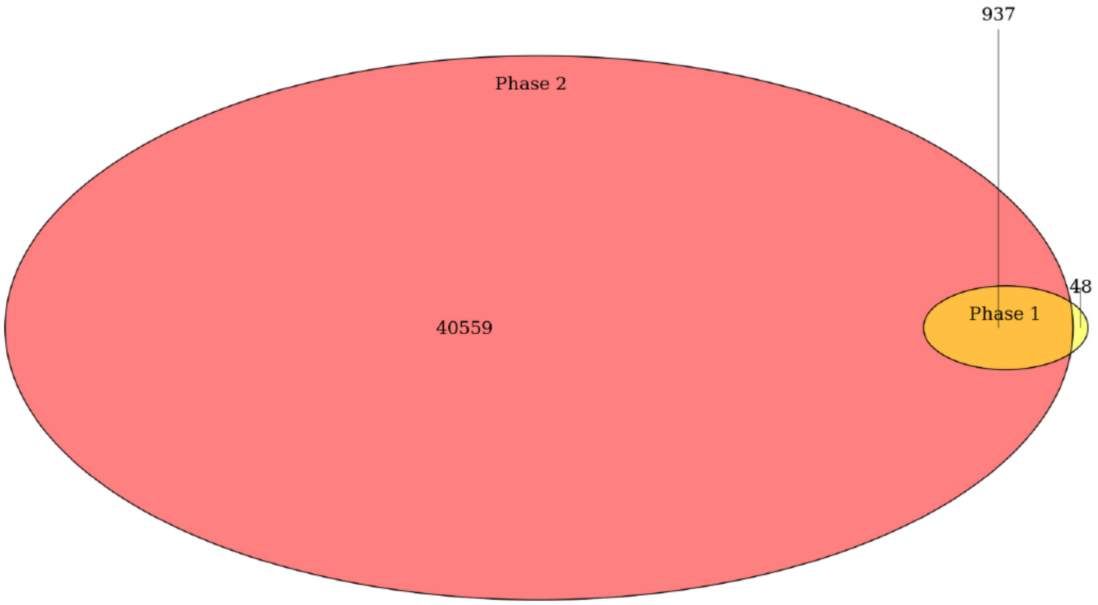

Supplementary Figure 4: Overlap in induced DMCs between Phase 1 and Phase 2, for all cytosine context. In yellow are the unique number of DMCs that were induced due to the Phase 1 memory effect but where not induced by the Phase 2 temperature regime. In orange are the DMCs which were induced both due to the Phase 1 and Phase 2 temperature regimes. In red are the DMCs induced solely due to the current Phase 2 temperature regimes.

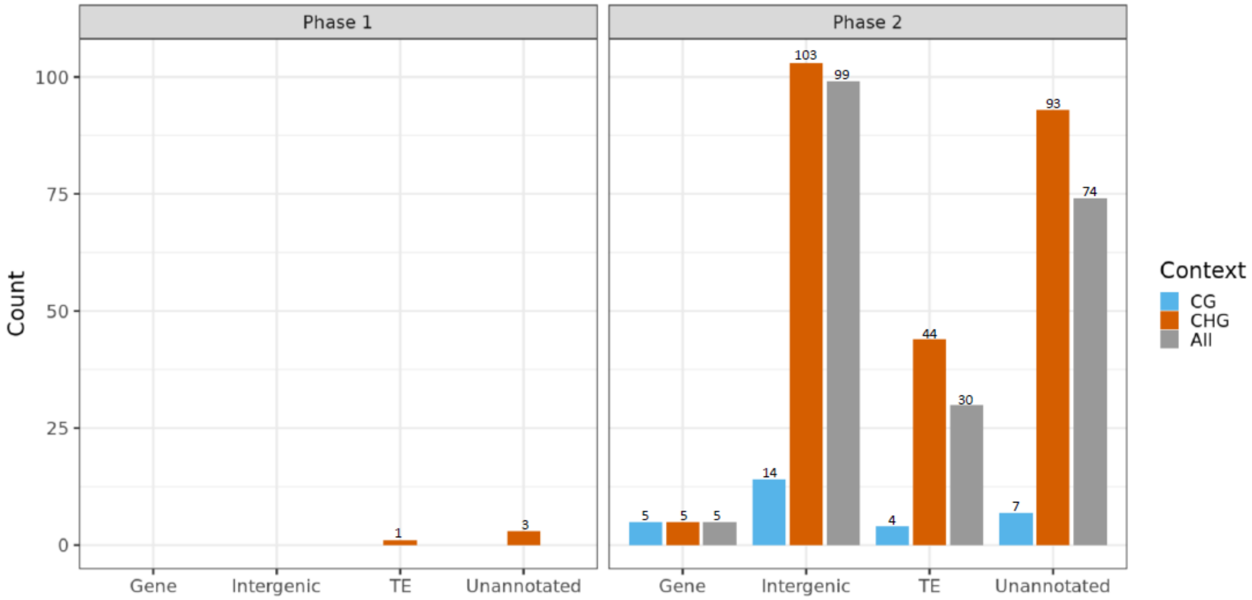

Supplementary Figure 5: Number of differentially methylated epiGBS loci detected. A locus was considered to be differentially methylated if it possessed 10 or more statistically significant DMCs, this for loci comprising of individual cytosine context DMCs (only CG, CHG or CHH DMCs) or loci for which a mixture of cytosine contexts defined a DMR (combination of CG, CHG and CHH DMCs). Differentially methylated epiGBS loci were found either within or near a gene (<1000 base pair), in intergenic regions, near or in a Transposable Elements or in unannotated regions. During Phase 1 lineages were exposed to either 4°C, 30°C or a weekly alternation of 24<>30°C. During Phase 2, lineages were then placed in a common environment of either 24°C or 30°C. No DMRs were detected in the CHH context and so was

MOLECULAR ECOLOGY

Supplementary Table 1: Meta-data file summarising the experimental group of Phase 1 and Phase 2 for each L.minor samples.

Sample Number	Species	Cultivar Number	Phase 1	Phase 2	Treatment group
1_1	*Lemna minor*	5500	24	24	t24-t24
2_1	*Lemna minor*	5500	24	24	t24-t24
3_1	*Lemna minor*	5500	24	24	t24-t24
4_1	*Lemna minor*	5500	24	24	t24-t24
5_1	*Lemna minor*	5500	24	24	t24-t24
6_1	*Lemna minor*	5500	24	24	t24-t24
7_1	*Lemna minor*	5500	24	24	t24-t24
8_1	*Lemna minor*	5500	24	24	t24-t24
9_1	*Lemna minor*	5500	24	24	t24-t24
10_1	*Lemna minor*	5500	24	24	t24-t24
11_1	*Lemna minor*	5500	24	24	t24-t24
12_1	*Lemna minor*	5500	24	24	t24-t24
13_1	*Lemna minor*	5500	24	24	t24-t24
14_1	*Lemna minor*	5500	24	24	t24-t24
15_1	*Lemna minor*	5500	24	24	t24-t24
16_1	*Lemna minor*	5500	24	24	t24-t24
17_1	*Lemna minor*	5500	30	24	t30-t24
18_1	*Lemna minor*	5500	30	24	t30-t24
19_1	*Lemna minor*	5500	30	24	t30-t24
20_1	*Lemna minor*	5500	30	24	t30-t24
22_1	*Lemna minor*	5500	30	24	t30-t24
23_1	*Lemna minor*	5500	30	24	t30-t24
24_1	*Lemna minor*	5500	30	24	t30-t24
25_1	*Lemna minor*	5500	30	24	t30-t24
26_1	*Lemna minor*	5500	30	24	t30-t24
27_1	*Lemna minor*	5500	30	24	t30-t24
28_1	*Lemna minor*	5500	30	24	t30-t24
30_1	*Lemna minor*	5500	30	24	t30-t24
33_1	*Lemna minor*	5500	tMix	24	tMix-t24
35_1	*Lemna minor*	5500	tMix	24	tMix-t24
36_1	*Lemna minor*	5500	tMix	24	tMix-t24
37_1	*Lemna minor*	5500	tMix	24	tMix-t24
38_1	*Lemna minor*	5500	tMix	24	tMix-t24
39_1	*Lemna minor*	5500	tMix	24	tMix-t24
40_1	*Lemna minor*	5500	tMix	24	tMix-t24
41_1	*Lemna minor*	5500	tMix	24	tMix-t24
42_1	*Lemna minor*	5500	tMix	24	tMix-t24
43_1	*Lemna minor*	5500	tMix	24	tMix-t24
44_1	*Lemna minor*	5500	tMix	24	tMix-t24
45_1	*Lemna minor*	5500	tMix	24	tMix-t24
46_1	*Lemna minor*	5500	tMix	24	tMix-t24

47_1	*Lemna minor*	5500	tMix	24	tMix-t24
48_1	*Lemna minor*	5500	tMix	24	tMix-t24
1_2	*Lemna minor*	5500	24	30	t30-t24
2_2	*Lemna minor*	5500	24	30	t30-t24
3_2	*Lemna minor*	5500	24	30	t30-t24
4_2	*Lemna minor*	5500	24	30	t30-t24
5_2	*Lemna minor*	5500	24	30	t30-t24
6_2	*Lemna minor*	5500	24	30	t30-t24
7_2	*Lemna minor*	5500	24	30	t30-t24
9_2	*Lemna minor*	5500	24	30	t30-t24
10_2	*Lemna minor*	5500	24	30	t30-t24
11_2	*Lemna minor*	5500	24	30	t30-t24
12_2	*Lemna minor*	5500	24	30	t30-t24
13_2	*Lemna minor*	5500	24	30	t30-t24
14_2	*Lemna minor*	5500	24	30	t30-t24
16_2	*Lemna minor*	5500	24	30	t30-t24
17_2	*Lemna minor*	5500	30	30	t30-t30
19_2	*Lemna minor*	5500	30	30	t30-t30
18_2	*Lemna minor*	5500	30	30	t30-t30
20_2	*Lemna minor*	5500	30	30	t30-t30
22_2	*Lemna minor*	5500	30	30	t30-t30
23_2	*Lemna minor*	5500	30	30	t30-t30
24_2	*Lemna minor*	5500	30	30	t30-t30
25_2	*Lemna minor*	5500	30	30	t30-t30
26_2	*Lemna minor*	5500	30	30	t30-t30
27_2	*Lemna minor*	5500	30	30	t30-t30
28_2	*Lemna minor*	5500	30	30	t30-t30
30_2	*Lemna minor*	5500	30	30	t30-t30
33_2	*Lemna minor*	5500	tMix	30	tMix-t30
35_2	*Lemna minor*	5500	tMix	30	tMix-t30
37_2	*Lemna minor*	5500	tMix	30	tMix-t30
38_2	*Lemna minor*	5500	tMix	30	tMix-t30
39_2	*Lemna minor*	5500	tMix	30	tMix-t30
40_2	*Lemna minor*	5500	tMix	30	tMix-t30
41_2	*Lemna minor*	5500	tMix	30	tMix-t30
43_2	*Lemna minor*	5500	tMix	30	tMix-t30
44_2	*Lemna minor*	5500	tMix	30	tMix-t30
45_2	*Lemna minor*	5500	tMix	30	tMix-t30
47_2	*Lemna minor*	5500	tMix	30	tMix-t30
48_2	*Lemna minor*	5500	tMix	30	tMix-t30

MOLECULAR ECOLOGY

Supplementary Table 2: Annotation of Differentially Methylated Regions landing in or near a gene. Only Phase 2 DMRs were found.

Cytosine Context	L. Minor ID	Gene Name	Other Name	GO Biological Process	GO Cellular Component	GO Molecular Function
CG	Lminor_004568	MED33A	AT3G23590, MED5A, REF4-RELATED 1, RFR1	Regulation of phenylpropanoid metabolic process	Mediator complex	
CG	Lminor_007868	PSBT	PSBTC	Photosynthesis	Photosystem II reaction centre	Chlorophyll binding
CG	Lminor_000410	Protein unknown function				
CG	Lminor_012894	EXPA8	ATEXP8, ATHEXP, ALPHA 1.11, EXP8	Plant-type cell wall loosening and wall organisation; responds to herbicide		
CG	Lminor_018952	CHI-B	THCHIB, HCHIB, PATHOGENESIS-RELATED 3, PR3	Defence response to fungus, jasmonic acid and ethylene-dependent systemic resistance ethylene mediated signaling pathway	Extracellular region	
CHG	Lminor_010295	SDR1	ABA2, ATABA2, ATSDR1, GIN1, ISI4, SIS4, SRE1	Abscisic acid biosynthesis process, proline biosynthesis process. Sugar mediated signalling pathway	Cytosol	Enables alcohol dehydrogensae (NAD+) activity; Identical protein binding; Xanthoxin dehydrogenase activity
CHG	Lminor_004914	SBE3				
CHG	Lminor_000440	Protein unknown function				
CHG	Lminor_013271	At3g09310		Membrane protein insertion efficiency factor		
CHG	Lminor_020172	UTG85A2	ATUGT85A2	Carboxylic acid metabolic process	Nucleus, chloroplast stroma and mitochondrion	Enables UDP-glycosyltranserfase activity
All	Lminor_006183	BFA2	At4g30825	DNA repair; Regulation of cyclin-depend protein serine/theorine kinase activity; regulation of transcription by RNA polymerase II	Transcription factor TFIIH-holo complex	Enables DNA binding, mRNA binding
All	Lminor_014961	BG2	ATBG2, ATPR2, BGL2, GNS2, PR-2, PR2	Responds to cold; Systemic acquired resistance; Carbohydrate metabolic process	Anchored component in plasma membrane.	Cellulase activity, protein binding
All	Lminor_016584	Protein unknown function				
All	Lminor_014151	NAR1	GOLLUM	Response to oxygen levels.	Cytosol and nucleus	

MOLECULAR ECOLOGY

Supplementary Table 3: Main proteins involved in the DNA methylation pathway and whose protein sequences were blasted against the transcriptome of L.minor. Protein queried refers to the NCBI reference sequence, Protein ID to the protein name and Length_protein to the sequence length of the targeted protein. HSP is the High-scoring Segment Pairs, with each HSP covering a specific length of the targeted protein. Coverage of each HSP is given in percentage with Per_ID refers to the percentage identity of each HSP. Closest_genomic_feature is the

Protein_Queried	Protein_ID	Length_Protein	HSP#	Length_Covered	Coverage (%)	E-value	Per_ID (%)	Closest_genomic_feature	Lemna_chr
NP_199727.1	MET1	1534	1	998	65.1	0	47.0	MET1B: DNA (cytosine-5)-methyltransferase 1B (Oryza sativa subsp. japonica)	Lminor_contig3109
			2	160	10.4	1e-67	57.5		
			3	103	6.7	1e-38	73.7		
			4	225	14.7	6e-70	44.0		
			5	68	4.4	6e-70	88.2		
NP_171612.1	DCL1	1909	1	403	21.1	0	71.4	DCL1: Endoribonuclease Dicer homolog 1 (Oryza sativa subsp. japonica)	Lminor_contig2744
			2	217	14.2	7e-102	60.5		
			3	270	14.1	0	63.3		
			4	231	12.1	2e-67	61.4		
			5	123	6.4	1e-122	63.4		
			6	73	3.8	1e-33	91.7		
			7	80	4.2	1e-122	78.7		
			8	341	17.9	1.e-47	36.3		
			9	61	3.2	6e-25	81.9		
			10	60	3.1	1e-122	83.3		
NP_189978.1	DCL3	1531	1	74	4.8	9e-96	52.7	DCL3A: Endoribonuclease Dicer homolog 3a (Oryza sativa subsp. japonica)	Lminor_contig3607
			2	45	2.9	2e-13	68.8		
			3	74	4.8	9e-96	50.0		
			4	130	8.5	3e-11	33.7		
			5	289	18.9	2e-39	33.5		
			6	149	9.7	9e-36	53.6		
			7	111	7.3	1e-24	50.4		
			8	70	4.6	9e-96	68.5		
			1	109	11.8	0	35.7		
			2	50	5.4	7e-5	46.0		
NP_565633.1	AGO4	924	3	25	2.7	0	68.0	AGO4A: Protein argonaute 4A (Oryza sativa subsp. japonica)	Lminor_contig4688
			4	449	48.6	0	35.1		
			5	186	20.1	0	41.9		
			6	159	17.2	0	40.2		
			7	134	14.5	0	41.0		
			8	59	6.4	0	67.7		
NP_177135.1	CMT3	839	1	87	10.4	6e-11	39.0	CMT3: DNA (cytosine-5)-methyltransferase CMT3 (Arabidopsis thaliana)	Lminor_contig6464
			2	24	2.9	2e-22	66.6		
			3	100	11.9	4e-41	67.0		
			4	27	3.2	4e-41	55.5		
			5	185	22.1	2e-22	35.1		
			6	151	18	2e-15	39.0		
			7	43	5.1	2e-12	67.4		
			8	98	11.7	2e-5	27.5		
NP_197042.2	DRM1	624	1	350	56.1	2e-41	60.8	DRM1: DNA (cytosine-5)-methyltransferase DRM1 (Arabidopsis thaliana)	Lminor_contig5882
NP_195305.2	NRPB1	1839	1	1055	57.4	0	81.4	NRPB1: DNA-directed RNA polymerase II subunit 1 (Arabidopsis thaliana)	Lminor_contig4091
			2	187	10.2	4e-154	82.8		
			3	156	8.5	4e-154	78.2		
			4	150	8.2	2e-54	67.3		
NP_193637.2	CMT1	791	1	91	11.5	2e-64	43.9	CMT1: DNA (cytosine-5)-methyltransferase CMT1 (Arabidopsis thaliana)	Lminor_contig6464
			2	35	4.4	2e-11	65.7		
			3	153	19.3	6e-5	38.5		
			4	86	10.9	2e-64	33.7		
			5	23	2.9	2e-11	60.8		
			6	25	3.2	1e-15	64.0		
			7	28	3.5	2e-5	50.0		
			8	26	3.3	2e-5	53.8		
			9	21	2.7	2e-64	51.9		
			10	27	3.4	2e-64	44.4		

MOLECULAR ECOLOGY

**NP_188907.2**	RDM1	163	1	123	75.5	8e-38	55.2	RDM1: Protein RDM1 (Arabidopsis thaliana)	Lminor_contig5478
**NP_196966.2**	DRM2	626	1	495	79.1	2e-153	39.9	DRM2: DNA (cytosine-5)-methyltransferase DRM2 (Arabidopsis thaliana)	Lminor_contig371
**NP_172932.1**	RDR1	1107	1	174	15.7	0	38.5	RDR1: RNA-dependent RNA polymerase 1 (Arabidopsis thaliana)	Lminor_contig3790
			2	171	15.4	0	37.4		
			3	640	57.8	0	56.8		
			4	349	31.5	0	56.4		
			5	265	23.9	0	58.4		
			6	230	20.8	0	56.0		
			7	223	20.1	0	65.4		
			8	275	24.8	0	46.9		
